# The combination of arsenic, interferon-alpha, and zidovudine restores an “immunocompetent-like” micro-environment in patients with adult T-cell leukemia lymphoma

**DOI:** 10.1186/1742-4690-11-S1-O4

**Published:** 2014-01-07

**Authors:** Ghada Kchour, SA Rahim Rezaee, Reza Farid, Akram Ghantous, Houshang Rafatpana, Mahdi Tarhini, Mohamad-Mehdi Kooshyar, Hiba El Hajj, Fadwa Berry, Roudaina Nasser, Abbas Shirdel, Zeina Dassouki, Mohamad Ezzedine, Hossein Rahimi, Ardeshir Ghavamzadeh, Hugues de Thé, Olivier Hermine, Mahmoud Mahmoudi, Ali Bazarbachi

**Affiliations:** 1 Department of Biology , Faculty of Sciences, Lebanese University , Hadath,Lebanon; 2 Microbiology and Virology Research Center, Bu-Ali Research institute, Mashhad University of Medical Sciences, Mashhad, Iran; 3 Immunology Research Centre Bu-Ali Research Institute, Mashhad University of Medical Sciences, Mashhad, Iran; 4 Lebanese American University, School of Arts and Sciences, Lebanon; 5 Islamic University, Faculty of Nursing Sciences, Lebanon; 6 Department of Internal Medicine, Mashhad University of Medical Sciences, Mashhad, Iran; 7 Department of Internal Medicine, American University of Beirut, Beirut, Lebanon; 8 Tehran University of Medical Sciences, Tehran, Iran; 9 INSERM UMR 944 and CNRS UMR 7212, Hôpital Saint Louis, Paris, France; 10 CNRS UMR 8147, Hôpital Necker, Paris, France

## 

HTLV-I associated adult T-cell leukemia/lymphoma (ATL) carries a dismal prognosis due to chemo-resistance and immuno-compromised micro-environment. The combination of zidovudine and interferon-alpha (IFN) significantly improved survival in ATL. Promising results were reported by adding arsenic trioxide to zidovudine and IFN. Here we assessed Th1/Th2/T_reg_ cytokine gene expression profiles in 16 ATL patients before and 30 days after treatment with arsenic/IFN/zidovudine, in comparison with HTLV-I healthy carriers and sero-negative blood donors. ATL patients at diagnosis displayed a T_reg_/Th2 cytokine profile with significantly elevated transcript levels of Foxp3, interleukin-10 (IL-10), and IL-4 and had a reduced Th1 profile evidenced by decreased transcript levels of interferon-γ (IFN-γ) and IL-2. Most patients (15/16) responded, with CD4^+^CD25^+^ cells significantly decreasing after therapy, paralleled by decreases in Foxp3 transcript. Importantly, arsenic/IFN/zidovudine therapy sharply diminished IL-10 transcript and serum levels concomittant with decrease in IL-4 and increases in IFN-γ and IL-2 mRNA, whether or not values were adjusted to the percentage of CD4^+^CD25^+^ cells. The observed shift from a T_reg_/Th2 phenotype before treatment toward a Th1 phenotype after treatment with arsenic/IFN/zidovudine may play an important role in restoring an immuno-competent micro-environment, which enhances the eradication of ATL cells and the prevention of opportunistic infections.

